# Efficacy of acupuncture-related therapies for gastroesophageal reflux-related chronic cough: a systematic review and meta-analysis

**DOI:** 10.3389/fmed.2026.1712003

**Published:** 2026-03-04

**Authors:** Tae-Young Choi, Lin Ang, Myeong Soo Lee

**Affiliations:** Korean Medicine Science Research Division, Korea Institute of Oriental Medicine, Daejeon, Republic of Korea

**Keywords:** acupuncture, chronic cough, gastroesophageal reflux disease, meta-analysis, quality of life, randomized controlled trial

## Abstract

**Background:**

Gastroesophageal reflux disease (GERD) may present as chronic cough, known as GERD-related chronic cough (GERC). Conventional treatment, including proton pump inhibitors, is often suboptimal. Acupuncture has been proposed as a complementary therapy, however, its clinical effectiveness for GERC remains unclear. This study aimed to evaluate the efficacy and safety of acupuncture-related therapies for GERC.

**Methods:**

We systematically searched 11 international and regional databases up to June 2025 for randomized controlled trials (RCTs) on acupuncture for GERC. Primary outcomes were daytime and nighttime cough symptom scores; secondary outcomes included the Leicester Cough Questionnaire (LCQ) score and total effective rate (TER). A random-effects model was used for meta-analysis. Risk of bias was assessed with RoB 2, and certainty of evidence with GRADE.

**Results:**

Five RCTs involving 390 participants were identified. Compared with Western medicine alone, acupuncture significantly reduced daytime (MD = −0.41, 95% CI [−0.75, −0.07]) and nighttime cough scores (MD = −0.38, 95% CI [−0.59, −0.17]). LCQ scores improved (MD = 2.29, 95% CI [1.99, 2.60], *p* < 0.00001), and TER was higher in the acupuncture group (RR = 1.13, 95% CI [1.01, 1.27]). No serious adverse events were reported. The overall risk of bias was moderate, mainly due to blinding and allocation limitations.

**Conclusion:**

Acupuncture may be a safe and effective complementary therapy for GERC, improving cough symptoms and quality of life. However, the current evidence is limited; larger, high-quality RCTs with standardized protocols are warranted.

**Systematic review registration:**

https://www.crd.york.ac.uk/prospero/display_record.php?RecordID=627037, Identifier CRD42024627037.

## 1 Introduction

Gastroesophageal reflux disease (GERD) is a chronic condition characterized by the retrograde flow of gastric contents into the esophagus, leading to classic symptoms such as heartburn and regurgitation. It can also present with atypical symptoms, including a chronic cough lasting more than 8 weeks, a condition known as GERD-related chronic cough (GERC). GERC accounts for approximately 25–40% of chronic cough cases in clinical settings (1).

The clinical features of GERC vary widely. These variations depend on factors such as the timing and context of the cough (e.g., nocturnal or postprandial), the presence or absence of classic GERD symptoms, and the individual’s response to acid-suppressive therapy. Notably, up to 75% of patients with GERD-induced cough present without heartburn or regurgitation, making diagnosis particularly challenging (2–5). In such cases, diagnostic confirmation typically requires a combination of clinical evaluation and objective tests such as 24-h esophageal pH monitoring (6). Moreover, empirical treatment with proton pump inhibitors (PPIs) is frequently used as a diagnostic tool (7). Nevertheless, many patients are misdiagnosed with respiratory conditions during the early stages and consequently receive inadequate treatment (2, 3).

Despite PPIs being the mainstay of therapy for GERD, their effectiveness in treating GERC is inconsistent, especially in cases involving non-acid reflux or esophageal hypersensitivity (8). These limitations have prompted interest in alternative and complementary therapies that target both reflux and cough pathways.

Acupuncture, a traditional therapeutic modality widely used in East Asian medicine, has shown promise in alleviating GERD symptoms and associated cough through multiple mechanisms. These include modulation of gastrointestinal motility, regulation of vagal tone, inhibition of acid secretion, and enhancement of esophageal sensory thresholds. Notably, acupuncture may strengthen lower esophageal sphincter (LES) tone and reduce reflux of both acid and bile (9, 10). Specific acupoints such as *Zusanli* (ST36), *Neiguan* (PC6), and *Zhongwan* (CV12) have been implicated in the regulation of digestive and respiratory function. Several clinical studies have reported beneficial effects of acupuncture in reducing GERD symptoms and improving cough-related quality of life (11).

Despite prior research and reviews exploring the effects of acupuncture on GERD, high-quality evidence specifically tailored to GERC patients remains limited. Therefore, this systematic review and meta-analysis aims to synthesize the current evidence from randomized controlled trials (RCTs) to comprehensively evaluate the efficacy and safety of acupuncture in the management of GERC.

## 2 Methods

### 2.1 Study design

This systematic review and meta-analysis were prepared following the principles of PRISMA guideline (12) (Supplementary File 5). The study protocol was pre-registered in the International Prospective Register of Systematic Reviews (PROSPERO) (13), with the registration number CRD42024627037 (https://www.crd.york.ac.uk/prospero/display_record.php?RecordID=627037).

### 2.2 Searches strategy

A comprehensive and systematic literature search was conducted across the following databases: PubMed, EMBASE, Cochrane Central Register of Controlled Trials (CENTRAL), China National Knowledge Infrastructure (CNKI), Wanfang, KoreaMed, Oriental Medicine Advanced Search Integrated System (OASIS), DBpia, Korean Medical Database (KMbase), Research Information Service System (RISS), and Korean Studies Information Services System (KISS). All relevant studies published from the inception of each database up to June 2025.

The search strategy utilized a combination of Medical Subject Headings (MeSH) and relevant keywords, including: (“Gastroesophageal Reflux” OR “GERD” OR “Gastric Acid Reflux” OR “Gastroesophageal Reflux Disease”) AND (“Cough”) AND (“Acupuncture” OR “Electroacupuncture”) AND (“Randomized Controlled Trial” OR “RCT”), with adjustments made for Korean and Chinese databases (Supplementary Files 1, 2). Manual searching of references and grey literature was also conducted to identify additional eligible studies.

### 2.3 Eligibility criteria

#### 2.3.1 Population

Adults aged 18–70 years diagnosed with GERC, based on either clinical evaluation or objective diagnostic criteria, were included.

#### 2.3.2 Intervention

Studies involving acupuncture-related therapies, including acupuncture and closely related TCM-based modalities (e.g., moxibustion, acupressure, and acupoint patching), used either as monotherapy or as an adjunct to conventional Western medicine (WM), were eligible for inclusion. Surgical interventions, including antireflux surgery such as fundoplication, were excluded because this review focused on non-surgical therapeutic approaches. Although antireflux surgery represents an established invasive treatment with distinct clinical indications and mechanisms and is known to effectively control GERC, the present review aimed to evaluate acupuncture-related therapies as non-surgical complementary or alternative treatment options.

#### 2.3.3 Comparator

Control groups received Western medical treatments such as proton pump inhibitors (PPIs) or antacids.

#### 2.3.4 Outcomes

The primary outcome was cough symptom scores, assessed during both daytime and nighttime, while secondary outcomes included the Leicester Cough Questionnaire (LCQ), Traditional Chinese Medicine (TCM) symptom scores (such as heartburn, regurgitation, and chest or throat discomfort), the Reflux Diagnostic Questionnaire (RDQ), and overall effectiveness rate.

#### 2.3.5 Study types

RCTs evaluating acupuncture or related therapies [including manual acupuncture (MA) and electroacupuncture (EA)] for the treatment of GERC were eligible, with no restrictions on language, publication status, or duration of treatment. Studies were excluded if they were non-RCTs, such as observational studies, case reports, animal studies, reviews; if they used multiple combined acupuncture-related modalities concurrently, such as acupuncture plus other TCM intervention; or were duplicate publications or trials with insufficient data for analysis.

### 2.4 Study selection and data extraction

The eligibility screening of all identified articles, including their titles, abstracts, and full texts, was performed by two independent reviewers (TYC and JHJ). Conflicts in screening decisions were resolved via discussion or by consultation with a third reviewer (MSL). We extracted a standardized set of data from each study, which encompassed: study characteristics (authors, year, country), sample size, participant demographics, diagnostic criteria, details of interventions and comparators, acupuncture protocol (acupoints, frequency, duration), outcome measures, and adverse events.

### 2.5 Risk of bias assessment

The methodological quality of included RCTs was assessed independently by two reviewers (LA and JHJ) using the Cochrane Risk of Bias version 2 (RoB 2) tool (14). Any discrepancies were resolved by consensus among all authors. The following domains were evaluated: randomization process, deviations from intended interventions, missing outcome data, measurement of the outcome, and selection of the reported result. The risk of bias for each domain was categorized as “low,” “some concerns,” or “high.”

### 2.6 Statistical analysis

Statistical analysis was performed using Review Manager (RevMan) version 5.4.1. Mean differences (MD) and 95% confidence intervals (CI) were computed for continuous outcomes, whereas risk ratios (RR) and 95% CI were used for dichotomous outcomes. Given the anticipated clinical and methodological variability, a random-effects model was applied. Heterogeneity was measured by the *I*^2^ statistic, where low, moderate, and high heterogeneity corresponded to thresholds of 25, 50, and 75%. To evaluate for potential publication bias, funnel plots were created when 10 or more studies were available for a specific outcome. Statistical significance was set at *p* < 0.05. The certainty of evidence for the primary and main secondary outcomes was assessed using the GRADE approach (15).

### 2.7 Certainty of evidence assessment

The certainty of evidence for each primary and secondary outcome was determined using the GRADE (Grading of Recommendations Assessment, Development and Evaluation) framework. We examined domains including risk of bias, inconsistency, indirectness, imprecision, and publication bias. Two reviewers (TYC and AL) independently assessed the overall quality of evidence for each outcome, resolving any disagreements through a consensus process. The effect estimates and certainty ratings were summarized in a Summary of Findings (SoF) table.

## 3 Results

### 3.1 Study selection

**Figure 1:**
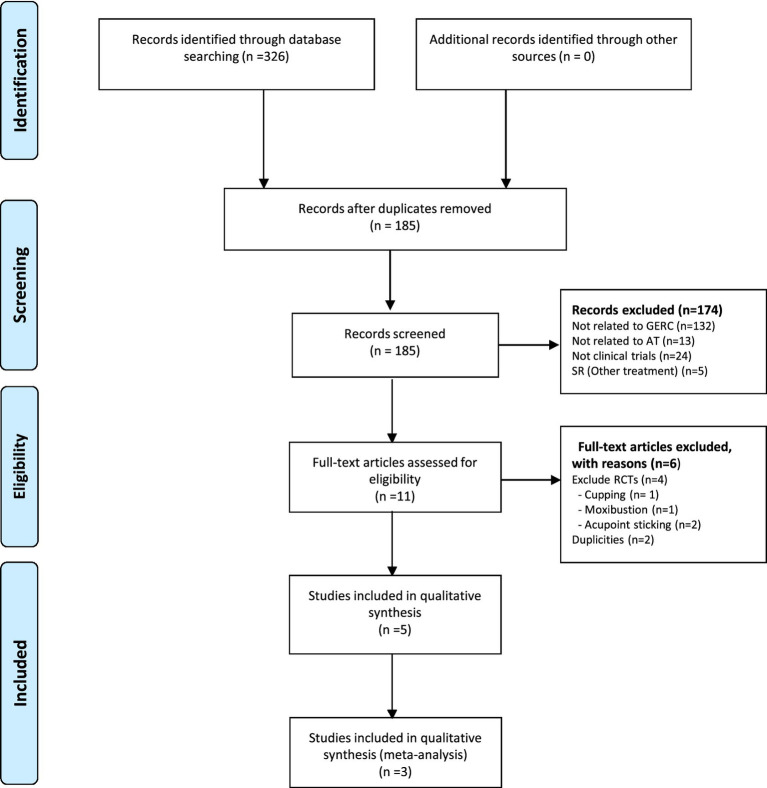
Flow chart of study selection. AT, acupuncture; SRs, systematic reviews; RCT, randomised clinical trial; GRDC, gastroesophageal reflux disease cough.

From the database search, 326 records were retrieved. After the elimination of 141 duplicates, 185 records advanced to the screening phase. An initial screening of titles and abstracts resulted in the exclusion of 174 records. The remaining 11 full-text articles were evaluated for eligibility, leading to the exclusion of 6 for not meeting the intervention criteria (*n* = 4, Supplementary File 3) or being duplicates (*n* = 2). Ultimately, 5 RCTs were included, with 3 of them incorporated into the final meta-analysis (Figure 1) (16–18).

### 3.2 Study characteristics

The included RCTs, published between 2018 and 2020, involved 390 patients diagnosed with gastroesophageal reflux-related chronic cough (GERC). Sample sizes ranged from 30 to 51 participants per group. Among the included studies, three RCTs evaluate acupuncture administered either as a standalone therapy or as an adjunct to WM. The WM comparators consisted of proton pump inhibitors and prokinetic or antacid agents, including domperidone (10 mg three times daily), pantoprazole (40 mg once or twice daily), omeprazole (20 mg twice daily), esomeprazole (10 mg once daily), mosapride citrate (5 mg three times daily), and hydrotalcite (0.5 g three times daily). In contrast, the remaining two studies investigated acupuncture-related therapies, such as moxibustion, acupressure, and acupoint patching, administered either alone or in combination with WM. Control groups received WM alone. The treatment duration ranged from 4 to 8 weeks.

All studies utilized acupuncture protocols targeting the dorsal segment of the Governor Vessel (GV6–GV12), with some including non-acupoint stimulation (Supplementary File 4). The main outcome measures were cough symptom scores (daytime/nighttime), Leicester Cough Questionnaire (LCQ), Traditional Chinese Medicine (TCM) symptom scores, Reflux Diagnostic Questionnaire (RDQ), and total effective rate (TER). Study details are summarized in Table 1.

**Table 1:** Summary of included studies.

First author (year)	Sample size (T/C) age (T/C)	Treatment group (acupoints)	Control group	Duration (weeks)	Outcomes	Result
Zhao et al. (2018) (16)	51/51 38 ± 9/39 ± 8	AT (GV9, qd) + WM	WM (domperidone 10 mg, tid; pantoprazole 40 mg, qd)	8	1) Cough score (daytime) 2) Cough score (nighttime) 3) LCQ 4) TER	1) MD −0.75 [−2.50, 1.00], *p* = 0.40 2) MD −0.78 [−2.53, 0.97], *p* = 0.38 3) MD 2.47 [2.31, 2.63], *p* < 0.00001 4) RR 1.17 [1.01, 1.36], *p* = 0.04
Guo et al. (2020) (17)	45/45 44 ± 6/44 ± 6	AT (GV12-GV8, T4, T8, qod) + WM	WM (pantoprazole 40 mg, bid; mosapride citrate 5 mg, tid; hydrotalcite 0.5 g, tid)	8	1) Cough score (daytime) 2) Cough score (nighttime) 3) LCQ 4) TCM score 5) TER	1) MD −0.22 [−0.37, −0.07], *p* = 0.004 2) MD −0.26 [−0.39, −0.13], *p* = 0.0002 3) MD 2.01 [1.14, 2.88], *p* < 0.00001 4) MD −1.51 [−2.46, −0.56], *p* = 0.002 5) RR 1.08 [0.91, 1.28], *p* = 0.37
Gao et al. (2019) (18)	30/30 51 ± 14/53 ± 11	AT (GV12-GV6, T4, T8, T12, qod)	WM (omeprazole 20 mg, bid)	4	1) Cough score (daytime) 2) Cough score (nighttime) 3) LCQ 4) RDQ	1) MD −0.60 [−0.83, −0.37], *p* < 0.00001 2) MD −0.50 [−0.68, −0.32], *p* < 0.00001 3) MD 2.10 [1.75, 2.45], p < 0.00001 4) MD −6.40 [−7.89, −4.91], *p* < 0.0001
Li et al. (2019) (20)	39/39 37 ± 8/37 ± 6	Moxa (heat-sensitive point, PC6, ST34, ST36, CV12 SP4, CV8, BL20, BL21, LU7, LU5, qd) + WM	WM (omeprazole 20 mg, bid; mosapride 5 mg, tid)	2	1) CSS 2) RDQ 3) LCQ 4) TER	1) MD −0.49 [−0.74, −0.24], *p* = 0.0001 2) MD −2.19 [−4.42, 0.04], *p* = 0.05 3) MD 0.38 [−0.11, 0.87], *p* = 0.13 4) RR 2.34 [0.64, 8.59], *p* = 0.20
Li et al. (2022) (21)	30/30 49 ± 11/4 8 ± 11	Acupressure plus AP (BL20, BL21, BL17, GV1-GV28, EX-B2)	WM (esomeprazole 10 mg, qd)	8	1) Cough score 2) Reflux symptoms score 3) Esophageal mucosal damage score 4) QOL	1) MD 3.00 [2.60, 3.40], *p* < 0.0001 2) MD −0.37 [−0.64, −0.10], *p* = 0.008 3) MD −0.40 [−0.67, −0.13], *p* = 0.004 4) MD 3.00 [2.60, 3.40], *p* < 000001

Characteristics of the included randomized controlled trials evaluating acupuncture-related therapies for gastroesophageal reflux-related chronic cough (GERC). The table summarizes study design, sample size, participant characteristics, details of acupuncture or acupuncture-related interventions, Western medicine comparators (including specific drug names, dosages, and administration frequencies), treatment duration, outcome measures, and main findings.

AP, acupoint patching; AT, acupuncture; CSS, cough severity score; GERC, gastroesophageal reflux cough; LCQ, Leicester Cough Questionnaire; Moxa, moxibustion; qd, once a day; qod, once every other day; QOL, quality of life; RDQ, Reflux Diagnostic Questionnaire; TCM, Traditional Chinese Medicine; TER, total effective rate; tid, three times a day; WM, Western medicine. Description of each acupoints are presented in Supplementary File 4.

### 3.3 Meta-analysis results

#### 3.3.1 Daytime cough symptom score

**Figure 2:**
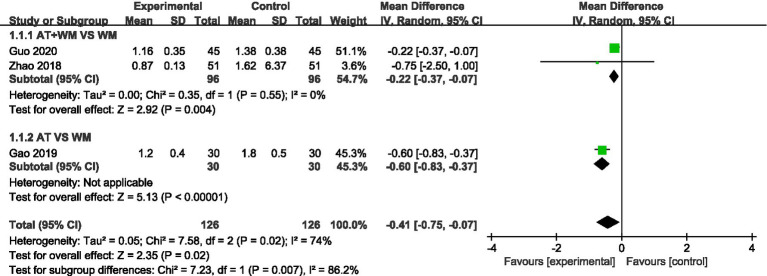
Forest plot of the meta-analysis for the daytime cough symptom score.

All three studies reported daytime cough scores. Meta-analysis showed a statistically significant improvement in the acupuncture group compared to controls (MD = −0.41, 95% CI [−0.75, −0.07], *p* = 0.02), with moderate heterogeneity (*I*^2^ = 74%) (Figure 2).

#### 3.3.2 Nighttime cough symptom score

**Figure 3:**
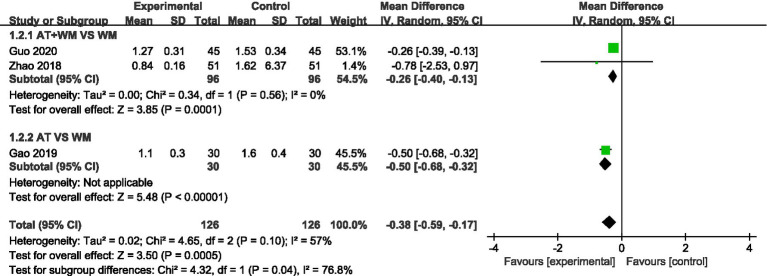
Forest plot of the meta-analysis for the nighttime cough symptom score.

Similarly, nighttime cough scores were significantly improved in the acupuncture group (MD = −0.38, 95% CI [−0.59, −0.17], *p* = 0.0005), with moderate heterogeneity (*I*^2^ = 57%) (Figure 3).

#### 3.3.3 Leicester Cough Questionnaire (LCQ)

**Figure 4:**
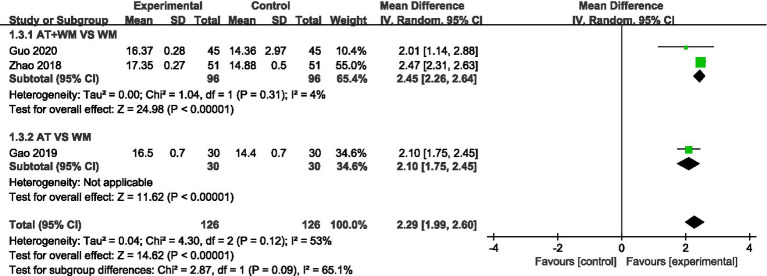
Forest plot of the meta-analysis for the Leicester Cough Questionnaire.

All studies reported LCQ scores (cough-related quality of life). The acupuncture group showed significantly greater improvements in LCQ total scores (MD = 2.29, 95% CI [1.99, 2.60], *p* < 0.00001), with moderate heterogeneity (*I*^2^ = 52%) (Figure 4).

#### 3.3.4 Total effective rate (TER)

**Figure 5:**
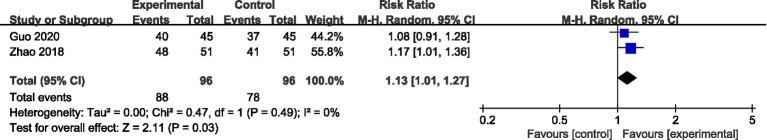
Forest plot of the meta-analysis for the total effective rate.

TER from all three studies demonstrated that acupuncture significantly increased the effective rate compared to control (RR = 1.13, 95% CI [1.01, 1.27], *p* = 0.03), with no observed heterogeneity (*I*^2^ = 0%) (Figure 5).

#### 3.3.5 Adverse events

No serious adverse events were reported in any of the included studies. Minor adverse events such as transient needling pain or local bruising were noted but did not lead to treatment discontinuation.

### 3.4 Risk of bias assessment

**Figure 6:**
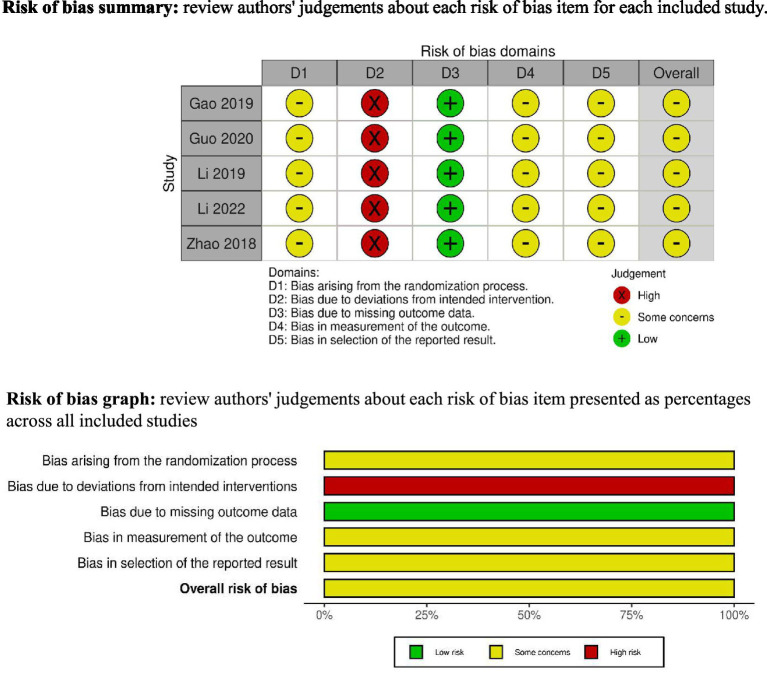
The figure represents the risk of bias assessment for the included studies.

The risk of bias was concerning or high across the included studies all trials reported appropriate random sequence generation. However, allocation concealment and blinding of outcome assessors were often not clearly described, leading to unclear or high risk in these domains. Risk of bias assessments are illustrated in Figure 6.

### 3.5 Certainty of evidence

The GRADE approach was applied to assess the certainty of the evidence for each key outcome. The overall certainty ranged from moderate to low, primarily due to limitations in study design, small sample sizes, and moderate heterogeneity in some outcomes. The detailed summary of findings is presented in Table 2.

**Table 2:** GRADE summary of findings for acupuncture-related therapies compared with Western medicine for gastroesophageal reflux-related chronic cough (GERC).

Outcome	No. of participants (studies)	Relative effect (95% CI)	Absolute effect	Certainty of evidence (GRADE)	Comments
Daytime cough score	252 (3 RCTs)	MD = −0.41 [−0.75, −0.07]	Mean decrease from 2.1 to 1.7	Moderate	Downgraded for risk of bias (unclear blinding) and inconsistency (*I*^2^ = 74%)^a^^,^^b^
Nighttime cough score	252 (3 RCTs)	MD = −0.38 [−0.59, −0.17]	Mean decrease from 1.9 to 1.5	Moderate	Downgraded for risk of bias (unclear blinding) and inconsistency (*I*^2^ = 57%)^a^^,^^b^
LCQ (QoL)	252 (3 RCTs)	MD = 2.29 [1.99, 2.60]	Mean increase from 12.3 to 14.6	Moderate	Downgraded for risk of bias and some imprecision^a^^,^^c^
Total effective rate (TER)	252 (3 RCTs)	RR = 1.13 [1.01, 1.27]	85% vs. 75%	Low	Downgraded for risk of bias and small sample size^a^^,^^d^

The table presents effect estimates and certainty of evidence ratings for primary and key secondary outcomes, including daytime and nighttime cough symptom scores, the Leicester Cough Questionnaire (LCQ), and total effective rate (TER), assessed using the GRADE approach.

GRADE, Grading of Recommendations, Assessment, Development and Evaluations; LCQ, Leicester Cough Questionnaire; MD, mean difference; QOL, quality of life; RCT, randomized controlled trial; RR, relative risk; TER, total effective rate.

Absolute effect: Calculated based on pooled baseline values in control group; risk of bias: All studies had unclear allocation concealment and limited blinding; inconsistency: moderate heterogeneity observed (*I*^2^ > 50%) for cough scores; imprecision: confidence intervals relatively wide for LCQ; publication bias: not assessed due to small number of studies (<10).

Absolute effects calculated using pooled means from control groups as baseline risk.

a Downgraded one level due to risk of bias: lack of blinding and allocation concealment across included trials.

b Downgraded one level due to inconsistency: moderate heterogeneity observed (*I*^2^ = 57–74%).

c Downgraded one level due to imprecision: wide confidence intervals.

d Downgraded two levels due to small sample sizes and imprecision in outcome estimates.

## 4 Discussion

This systematic review and meta-analysis represent one of the first comprehensive evaluations of the effectiveness of acupuncture related therapies for GERC. GERC represents a distinct extra-esophageal manifestation of GERD. Therefore, evidence from GERD-focused acupuncture research provides important contextual support for interpreting the present findings. In this context, the current meta-analysis demonstrates that acupuncture-related therapies may improve multiple clinical outcomes associated with GERC, including daytime and nighttime cough symptoms, quality of life (as measured by LCQ), total effective rate. Moreover, the meta-analysis demonstrated that acupuncture is effective both as a standalone treatment and when used in combination with conventional Western medicine. However, the findings should be interpreted with caution given the limited number and methodological quality of the included studies.

Our results align with prior meta-analysis evaluating the effectiveness of acupuncture for GERD. Previous review has reported that acupuncture is a potentially effective and safe treatment for GERD. However, they highlighted limitations such as small sample size and low methodological quality of the included studies (11). Additionally, the inclusion of a broad spectrum of GERD related symptoms in those studies limited the ability to isolate acupuncture’s effect on chronic cough specifically. This study addresses those gaps by focusing on chronic cough which is a distinct and clinically relevant symptom of GERD; thus offering a more targeted and meaningful evaluation.

Acupuncture is a core modality in TCM for managing GERC (19). It is believed to regulate qi and blood, harmonize organ function, and dredge meridians. Frequently used acupoints include *Neiguan* (PC6), *Zusanli* (ST36), and *Zhongwan* (CV12) for excess syndrome. Other commonly employed points include *Pishu* (BL20), *Weishu* (BL21), *Shenshu* (BL23), *Tanzhong* (CV17), *Quchi* (LI11), *Hegu* (LI4), *Taichong* (LR3), *Tianshu* (ST25), *Guanyuan* (CV4), and *Sanyinjiao* (SP6). Acupuncture points located along the dorsal Governor Vessel (GV) have also demonstrated promising therapeutic effects, often using balanced tonification and drainage techniques (Supplementary File 4).

Several individual clinical studies have further supported the beneficial effects of acupuncture and related TCM therapies for GERC. Zhao et al. (16) reported *Zhiyang* (GV9) in combination with pantoprazole sodium, and reported significant improvements in both cough and accompanying TCM symptoms compared to pantoprazole alone. Guo et al. (17) applied acupuncture along the Governor Vessel (GV12, GV11, GV10, GV9, GV8) and non-acupoints, in combination with proton pump inhibitors (PPIs), showing better symptom relief and lower TER compared to conventional triple therapy. Similarly, Gao and Bai (18) employed an extended range of GV points (GV12 through GV6) and reported superior outcomes in all clinical measures compared to omeprazole. Li et al. (20) found that heat-sensitive point moxibustion combined with PPIs markedly reduced cough and reflux symptoms, improved quality of life, and yielded a higher TER than PPIs alone. Li et al. (21) evaluated the “*Yishu Tiaoshu*” acupoint therapy, which integrates herbal mist application with back *shu*-point massage, in combination with conventional treatment, and observed significant improvements in cough and reflux symptoms, as well as quality of life. This therapy appeared particularly effective for patients with GERC characterized by the “adverse rising of stomach qi” pattern.

However, several limitations warrant consideration. The number of included trials was relatively small, with most studies featuring limited sample sizes, thereby reducing generalizability. All included trials were rated as high risk in the domain of deviations from intended interventions, primarily due to the lack of participant blinding, insufficient reporting of treatment adherence, and limited control of co-interventions. These factors may have introduced performance bias and potentially inflated effect estimates. The observed heterogeneity (*I*^2^ = 74%) may be attributable to differences in treatment duration, acupuncture frequency, and intervention type (acupuncture alone vs. acupuncture combined with Western medicine). However, due to the limited number of included studies, formal subgroup or sensitivity analyses were not statistically feasible. Methodological shortcomings, particularly insufficient allocation concealment, lack of blinding of outcome assessors, and inconsistently reported intervention protocols, may have introduced bias (22, 23). Moreover, all included studies were conducted in China, which may limit the external validity of our findings across different healthcare systems or cultural contexts.

Despite these limitations, this meta-analysis has notable strengths. First, we minimized publication bias by conducting a comprehensive and systematic search across multiple databases. Second, we included only RCTs, which provide the highest level of evidence for assessing intervention efficacy. Third, rigorous statistical methods were applied to synthesize data and assess the robustness of outcomes. Together, these approaches enhance the reliability of our findings.

Overall, our results provide preliminary but meaningful evidence supporting the use of acupuncture as a complementary treatment for GERC. To strengthen the evidence base, future research should include adequately powered, multicenter RCTs with standardized acupuncture protocols. Additional mechanistic studies and subgroup analyses, particularly among patients with PPI-refractory symptoms, are also warranted to further clarify acupuncture’s therapeutic potential.

## 5 Conclusion

This systematic review and meta-analysis provide preliminary but consistent evidence suggesting that acupuncture may serve as an effective and safe complementary treatment for GERC. Future high-quality studies are needed to confirm these findings and further explore the clinical role of acupuncture in the management of GERC.

## Data Availability

The datasets presented in this study can be found in online repositories. The names of the repository/repositories and accession number(s) can be found in the article/Supplementary material.
