# Supplementary material for: Efficacy of acupuncture-related therapies for gastroesophageal reflux-related chronic cough: a systematic review and meta-analysis
Source: Front Med (Lausanne). 2026 Mar 4;13:1712003. doi: 10.3389/fmed.2026.1712003 (PMC12996054; doi:10.3389/fmed.2026.1712003)
Supplement: Supplementary file 1 [file Table_1.DOCX]

**Supplementary Materials**

**Supplemental 1. The English search strategy takes PubMed as an example.**

#1. "Gastroesophageal Reflux" [Mesh] OR "Gastroesophageal reflux" [TW] OR "GERD" [TW] OR "Gastric Acid Reflux" [TW] OR "Gastro Esophageal Reflux Disease*" [TW]

#2. "Cough" [Mesh] OR "Cough" [TW]

#3. "Acupuncture Therapy"[Mesh] OR "Acupuncture"[Mesh] OR "Acupuncture Points"[Mesh] OR "Acupuncture Analgesia"[Mesh] OR "Acupuncture, Ear"[Mesh] OR (Acupunct*[TW] OR acupoint*[TW] OR electroacupunct*[TW] OR electro-acupunct*[TW] OR pharmacopuncture[TW] OR auriculotherap*[TW]

#4. #1 AND #2 AND #3

**Supplemental 2. The Chinese search strategy takes CNKI as an example.**

#1. (胃食管反流病 OR 胃酸反流 OR 反流性食管炎)

#2. (咳嗽 OR 慢性咳嗽 OR 反流性咳嗽))

#3. (针灸 OR 针刺 OR 电针 OR 耳针 OR 头针 OR 体针 OR 毫针)

#4. #1 AND #2 AND #3

**Supplemental 3. Exclusion list of RCTs for GERC.**

| **First Author (Year)** | **Intervention** | **Reason for exclusion** |
| --- | --- | --- |
| Fan (2018) [1] | Moxibustion plus CHM vs WM | Not AT treatment |
| Cheng (2021) [2] | Acupoint application plus CHM vs WM | Not AT treatment |
| Zhou (2023) [3] | Acupoint application plus CHM vs WM vs CHM | Not AT treatment |
| Xiao (2014) [4] | Cupping plus CHM vs WM | Not AT treatment |

AT: acupuncture; CHM: Chinese Herbal Medicine; WM: western medicine

**References**

1. Pan, X., B. Lu, and L. Yang, *Clinical observation of moxibustion combined with Banxia Xiexin Decoction in the treatment of gastroesophageal reflux-induced chronic cough.* Shanghai Journal of Acupuncture and Moxibustion, 2018. **37**(08): p. 883-886.

2. Cheng, N., Y. Song, and Y. Yang, *Clinical observation of Xuanfu Daizhe Decoction combined with acupoint application in the treatment of gastroesophageal reflux-induced chronic cough.* Guangming Journal of Chinese Medicine, 2021. **36**(24): p. 4114-4117.

3. Zhou, M., et al., *Clinical observation of Jianpi Xiaopi Decoction combined with acupoint application in the treatment of gastroesophageal reflux-induced chronic cough.* Journal of Guangzhou University of Chinese Medicine, 2023. **40**(06): p. 1376-1381.

4. Xiao, E. and Z. Luo, *Clinical observation on modified Banxia Xiexin Decoction combined with cupping therapy in the treatment of 40 cases of gastroesophageal reflux-induced chronic cough.* Chinese Journal of National and Folk Medicine, 2014. **23**(22): p. 64.

**Supplemental 4.** Acupoints used in the included studies

| **English Name (Pinyin)** | **Standard Acupoint Code** | **Key Features / Location Description** |
| --- | --- | --- |
| Geshu | BL17 | 1.5 cun lateral to the lower border of T7 spinous process |
| Pishu | BL20 | 1.5 cun lateral to the lower border of T11 spinous process |
| Weishu | BL21 | 1.5 cun lateral to the lower border of T12 spinous process |
| Shenque | CV8 | At the center of the umbilicus |
| Zhongwan | CV12 | 4 cun above the umbilicus on the midline of the abdomen |
| Mingmen | GV4 | Below L2 (2nd lumbar vertebra), on the midline of the spine |
| Zhiyang | GV9 | Below T7 (7th thoracic vertebra), on the midline of the spine |
| Zhongzhu | GV7 | Below T10 (10th thoracic vertebra), on the midline of the spine |
| Jinzhu | GV8 | Below T9 (9th thoracic vertebra), on the midline of the spine |
| Shenzhu | GV12 | Below T3 (3rd thoracic vertebra), on the midline of the spine |
| Dazhui | GV14 | Below C7 (7th cervical vertebra), on the midline of the spine |
| Chize | LU5 | At the cubital crease, on the radial side of the tendon of biceps brachii |
| Lieque | LU7 | 1.5 cun above the wrist crease on the radial side of the forearm, between the radius and brachioradialis |
| Gongsun | SP4 | On the medial side of the foot, in the hollow distal and inferior to the base of the first metatarsal bone |
| Liangqiu | ST34 | 2 cun above the patella, lateral side of the rectus femoris muscle |
| Zusanli | ST36 | 3 cun below the knee, lateral side of the tibialis anterior muscle |
| Neiguan | PC6 | On the inner forearm, 2 cun above the wrist crease, between the two tendons |
| Huatuojiaji | EX-B2 | 0.5 - 1 cun lateral to the lower border of the spinous processes of C1 to L5 vertebrae, bilateral location |

BL: Bladder; CV: Conception Vessel; EX-B2: Huatuojiaji; GV: Governor Vessel; LU; Lung; PC: Pericardium; SP: Spleen; ST: Stomach; GV (Du Meridian,): Points along the midline of the back; EX-B2 (Huatuojiaji)Extra points beside the spine used for neuro-musculoskeletal and visceral disorders; CV (Conception Vessel, Ren Mai): Points along the anterior midline of the body.
